# Optimization of Culture Conditions and Batch Process Control for the Augmented Production of Bacteriocin by *Bacillus* Species

**DOI:** 10.3390/microorganisms12040651

**Published:** 2024-03-25

**Authors:** Ahmed M. Elazzazy, Mona O. Mobarki, Afra M. Baghdadi, Noor M. Bataweel, Ahmed M. Al-Hejin

**Affiliations:** 1 Department of Biological Sciences, College of Science, University of Jeddah, P.O. Box 80327, Jeddah 21589, Saudi Arabia; 1900485@uj.edu.sa (M.O.M.); amboghdadi@uj.edu.sa (A.M.B.); 2 King Fahad Medical Research Center, King Abdulaziz University, P.O. Box 80216, Jeddah 21589, Saudi Arabia; no0ora.118@hotmail.com (N.M.B.); aalhejin@kau.edu.sa (A.M.A.-H.); 3 Department of Biological Sciences, Faculty of Science, King Abdulaziz University, P.O. Box 80216, Jeddah 21589, Saudi Arabia

**Keywords:** antimicrobial peptides, bacteriocin, rhizosphere, PCR, bioreactor, batch process, *Bacillus atrophaeus*, *Bacillus amyloliquefaciens*

## Abstract

The emergence of antibiotic-resistant microorganisms poses a significant threat to human health worldwide. Recent advances have led to the discovery of molecules with potent antimicrobial activity from environmental sources. In this study, fifteen bacterial isolates were obtained from agricultural and polluted soil samples collected from different areas of the cities of Jizan and Jeddah. These isolates were screened for antagonistic activity against a set of human pathogenic bacterial strains. The results showed that two *Bacillus* strains, identified as *Bacillus atrophaeus* and *Bacillus amyloliquefaciens* based on 16S rDNA, synthesized bacteriocin with strong antibacterial activity against Methicillin-resistant *Staphylococcus aureus* (MRSA) ATCC 33591, *Pseudomonas aeruginosa* ATCC 9027, *Salmonella typhimum* ATCC 14028, carbapenem-resistant *E. coli*, and MRSA 2. To optimize bacteriocin production, the effects of medium composition, incubation period, temperature, and pH were investigated. Nutrient broth and Mueller–Hinton broth were chosen as the optimal original media for bacteriocin production. The optimal incubation period, temperature, and pH were found to be 48 h at 37 °C and 7 pH in *Bacillus atrophaeus* and 72 h at 37 °C and 8 pH in *Bacillus amyloliquefaciens*. Batch cultures of *Bacillus atrophaeus* and *Bacillus amyloliquefaciens* were grown in a 10 L benchtop bioreactor, and pH control was found to significantly increase the production of bacteriocin by two-fold compared to uncontrolled conditions. The time course of growth, substrate consumption, pH, and enzyme production were investigated. This study demonstrates the potential of optimizing culture conditions and batch process control to enhance bacteriocin production by *Bacillus* spp.

## 1. Introduction

The medical community initially hailed antibiotics as groundbreaking treatments that would forever alter the course of medicine. However, despite this optimism, early reports of therapeutic shortcomings and emerging resistance to antibiotics have cast a shadow over their efficacy [1]. In 2021, multidrug-resistant and pandrug-resistant bacteria are ubiquitous globally, leading to increased mortality and morbidity rates as well as economic burdens [2]. Given the escalating crisis of antibiotic resistance, which is nearing pandemic proportions, novel antibiotics or alternative treatments are urgently needed. Compounds capable of eliminating pathogens, particularly hospital-acquired infections that often manifest multidrug resistance, are of special interest [3]. Antimicrobial peptides (AMPs), initially identified over two decades ago, have long offered promise as alternative therapies [4]. These naturally occurring molecules, which range from 6 to 100 amino acids in length, have persisted for millions of years without promoting resistance [5]. Produced endogenously by multicellular organisms, AMPs not only serve as integral components of the innate immune system but are also recognized as host defense peptides [6]. Their broad-spectrum activity encompasses antibacterial effects against a wide array of pathogens, including protozoans, viruses, and fungi [7,8,9]. Furthermore, AMPs exert their effects primarily through membrane disruption, owing to their amphipathic properties [10]. The term “bacteriocins” is used to describe a category of AMPs that are synthesized by bacteria [11]. Typically composed of between 12 and 50 amino acids, over 3257 different AMPs have been documented to date according to the Antimicrobial Peptide Database [12,13]. Various *Bacillus* species produce several types of bacteriocins, including *B. thuringiensis*, *B. thermoleovorans*, *B. licheniformis*, *B. cereus*, *B. amyloliquefaciens*, *B. subtilis*, and *B. coagulans* [14,15,16,17,18,19,20]. The utilization of *Bacillus* spp. in bacteriocin production is justified due to their ability to produce a diverse range of potent antimicrobial peptides. *Bacillus* species offer advantages such as robust growth characteristics, versatility in environmental conditions, and a history of safe use in industrial applications. They are suitable for large-scale fermentation processes and can be genetically engineered to enhance bacteriocin production. This makes *Bacillus* spp. preferred for industrial applications. Each of these *Bacillus* species exhibits distinct properties and mechanisms of action. Among the most notable are thuricin, kurstacin, and entomocins synthesized by *Bacillus thuringiensis*, which exhibit antimicrobial activity against both bacteria and insect pests [21]. *Bacillus subtilis* produces subtilin, a lantibiotic bacteriocin known for its potent antibacterial effects against Gram-positive bacteria [22]. Lichenicidin, a two-component bacteriocin from *Bacillus licheniformis*, demonstrates inhibitory activity against Gram-positive bacteria, including pathogens like *Staphylococcus aureus* [23]. Various bacteriocins, such as cerein and bacillomycin, are produced by *Bacillus cereus* strains, exerting antimicrobial effects against both Gram-positive and Gram-negative bacteria [24]. *Bacillus amyloliquefaciens* synthesizes amylocyclicin, a cyclic bacteriocin with broad-spectrum antimicrobial activity against foodborne pathogens [21,25].

Despite their remarkable attributes, the large-scale production of bacteriocins faces multiple challenges. Critical among these is the optimization of culture conditions and medium composition [26]. Consequently, this study aims to focus on optimizing the culture medium at shake flask levels and scaling up the cultivation of *B. atrophaeus* and *B. amyloliquefaciens* to a semi-industrial 10 L bioreactor to enhance bacteriocin yields. Through this research, we hope to contribute significantly to the pressing need for alternative antimicrobial agents that can effectively combat the growing threat of antibiotic resistance.

## 2. Materials and Methods

### 2.1. Chemicals and Reagents

The majority of bacteriological media utilized in this study were procured from Oxoid Co., Thermo Fisher Scientific, Basingstoke, UK, unless otherwise specified. Proteinase K and Dream Taq Green Master Mix 2X were obtained from Thermo Scientific Co., Waltham, MA, USA. Solvents were sourced from Sigma Co., Saint Louis, MO, USA. Lysozyme and agarose were acquired from Bio Basic Inc., Markham, ON, Canada. The DNA ladder (100 bp DNA Ladder H3 RTU—Ready-to-Use) was purchased from GeneDirex, Inc., Taoyuan, Taiwan. Universal primers specific for the 16S rDNA gene were synthesized by Bioneer Co., Daejeon, Republic of Korea.

### 2.2. Bacterial Strains

The preparation of the sample solution was as follows: Fifteen soil samples were gathered from the cities of Jeddah and Jizan in Saudi Arabia, each taken from a depth of 5–10 cm. At each site, two replicates were randomly collected. The soil samples were carefully transferred into clean containers using sterile techniques. To prepare the sample solution, 0.1 mL of each soil sample was serially diluted in sterilized distilled water containing 0.5% peptone. The diluted samples were then evenly spread onto Petri dishes filled with nutrient agar media. These plates were subsequently incubated at 37 °C for durations of 48 and 72 h. After incubation, distinct morphological colonies of *Bacillus* species were observed. These colonies were carefully isolated and maintained as pure cultures by storing them in MRS broth supplemented with 20% glycerol at −80 °C for future experimentation.

### 2.3. Characterization and Identification of Test Isolates

The bacterial isolates were identified through an examination of their morphological and microscopic features, following the procedures detailed in Bergey’s Manual [27]. Furthermore, to validate the identity of two strains, we utilized 16S rRNA sequencing. Genomic DNA extraction from all soil samples was performed using the method outlined by [28], with minor adjustments. Bacterial pellets were precipitated with low-speed centrifugation (Sigma, Osterode am Harz, Germany) at 5000× *g* for 10 min. A total of 200 μL of TES buffer and 20 μL of lysozyme (10 mg/mL) were added to the bacterial pellets and incubated at 37 °C for 20 min. A total of 10 mg/mL of proteinase K was added to each tube then incubated again at 37 °C for an extra 20 min, then the tubes were chilled in an ice bath for 5 min. A concentration of 4 M sodium acetate (250 μL) and an equal volume of chloroform–isoamyl (24:1) were added to each tube and gently mixed. The tubes were centrifuged at 14,000 rpm for 5 min, and the upper aqueous phase was transferred to a new sterile Eppendorf tube, and 3/4 or 1 *v*/*v* of isopropyl alcohol was added. The tubes were kept at −20 °C overnight. The tubes were centrifuged at 14,000 rpm for 10 min, and the upper layer was decanted. The DNA pellets were air-dried at room temperature then resuspended in 50 μL of distilled water. Ten microliters (10 µL) of genomic DNA (gDNA) was electrophoresed in 0.5% agarose gel in 1x Tris–Borate–EDTA (TBE) buffer (Tris base: 0.089 M (pH 8.3), Boric acid: 0.089 M, EDTA: 0.002 M) at 100 volts for 25 min and stained with ethidium bromide.

### 2.4. 16S rRNA Gene Amplification by PCR

DNA amplification was performed using PCR master mix (Thermo Scientific) following the manufacturer’s manual. A total of 10 ng of genomic DNA isolated from each strain was amplified using Universal 16S rDNA bacterial primers 27F (5′-AGAGTTTGATCCTGGCTCAG-3′) and 1492R (5′-AAGGAGGTGATCCAGCCGCA-3′). A negative control of 2 μL 0.1 × TE (Tris EDTA) was used. gDNAs were diluted 1:10, and 2 μL of each dilution was added to 25 μL of master mix containing 2 μL of (25 pmole) 16S rDNA primer mix. PCR amplification was conducted in a gradient thermocycler (Eppendorf, Hamburg, Germany) at 94 °C for 5 min, followed by 35 cycles of 45 s at 94 °C, 45 s at 57 °C, and 90 s at 72 °C, with a final extension step at 72 °C for 7 min. An aliquot of 10 μL of each PCR product was electrophoresed on a 1% agarose gel containing 0.5 μg/mL ethidium bromide and visualized on a UV transilluminator (Bio-rad, Hercules, CA, USA) to confirm the presence of a 1500 bp band. Purified PCR fragments were sequenced with both primers and compared with 16S rRNA gene sequences in a public database using BLAST.

### 2.5. Antimicrobial Susceptibility Test

Agar well diffusion methods were used to evaluate the susceptibility against the following tested pathogenic bacteria: *Pseudomonas aeruginosa* ATCC 9027, *Staphylococcus aureus* MRSA ATCC 33591, *Salmonella typhimurium* ATCC 14028, carbapenem-resistant *E. coli*, and MRSA 2, isolated from King Abdul-Aziz University hospital. These bacteria were obtained from the culture collection of the microbiology lab in King Fahd Medical Research Centre (KFMRC) at King Abdul-Aziz University (Jeddah, Saudi Arabia). Mueller–Hinton agar plates were inoculated with a standard inoculum (0.5 McFarland) of the test microorganisms. Then, four wells with a diameter of 8 mm were punched aseptically with a large tip, and 100 µL of cell-free supernatants (CFSs) were injected into each well. Finally, agar plates were incubated at 37 °C for 24, 48, and 72 h. After overnight incubation, the diameter of the clear zone around each well was measured in millimeters.

### 2.6. Evaluating the Influence of Incubation Duration, pH, and Temperature on Bacteriocin Synthesis

Incubation periods, pH, and thermal conditions are critical variables in the biosynthesis of bacteriocins. Both nutrient and Mueller–Hinton broths were inoculated using a sterilized loop containing *Bacillus* spp. isolates. These cultures were then subjected to various incubation periods of 4, 8, 16, 20, 24, 28, 32, 48, and 72 h and at temperatures of 35 °C, 37 °C, and 45 °C. Concurrently, the pH was modified across a range of 5.0, 6.5, 7.0, 8.0, and 9.0, utilizing 1 N NaOH and 1 N HCl, and incubated under shaking conditions. After incubation, cultures were centrifuged at 10,000 rpm for 30 min. to separate bacterial cells from the supernatant, which was then transferred to sterile tubes for further analysis. Bacteriocin activity was assessed against indicator strains like Methicillin-resistant Staphylococcus aureus (MRSA) using the well diffusion method.

### 2.7. Assessing the Impact of Varied Culture Media on Bacteriocin Efficacy

Six different culture media containing diverse carbon and nitrogen sources such as lactose, glucose, beef extract, yeast extract, and peptone were evaluated to ascertain their efficiency in promoting bacteriocin production in *Bacillus* spp. The media under scrutiny were Mueller–Hinton broth (MHB), Luria broth (LB), Tryptic Soy broth (TSB), Brain Heart Infusion (BHI), Soybean Casein Digest broth, nutrient broth (NB), and Mineral Salt Media (MS). The level of bacteriocin production was quantified through optical density measurements using a spectrophotometer (Perkin Elmer, Lamda, Kowloon, Hong Kong), and the efficacy was tested against MRSA indicator strains.

### 2.8. Bioreactor Cultivation Procedures

Bioreactor cultivation was conducted using a 10 L benchtop bioreactor (New Brunswick, NJ, USA) equipped with a Rushton turbine impeller. Key parameters, including dissolved oxygen (DO), temperature, pH, agitation speed, and foam control, were meticulously controlled throughout the fermentation process to optimize bacteriocin production.

#### 2.8.1. Dissolved Oxygen (DO) Control

Oxygen was manually introduced into the culture medium at a constant rate of 100% (1.0 vvm) via a flow meter to maintain optimal DO levels. The dissolved oxygen levels were regulated between 0 and 100% throughout the fermentation process to ensure adequate oxygenation for microbial growth and bacteriocin production.

#### 2.8.2. Temperature Control

The temperature was consistently maintained at 37 °C throughout the cultivation period to create an optimal environment for bacterial growth and metabolite production.

#### 2.8.3. pH Control

pH was continuously monitored throughout the fermentation process using a glass electrode (Ingold). To maintain an optimal pH range for bacteriocin production, adjustments were made either manually or through automated pH control systems. Manual adjustments involved periodic monitoring of the pH using a glass electrode (Ingold) and adding predetermined volumes of appropriate buffer solutions, such as phosphate buffers (e.g., potassium phosphate buffer for pH 6–8) or acetate buffers (e.g., sodium acetate buffer for pH 4–5), as needed. The selection of buffer solutions was based on their capacity to effectively stabilize pH without adverse effects on bacterial growth or bacteriocin production. This manual pH management approach ensured precise control over fermentation conditions, thereby enhancing bacteriocin synthesis. Notably, while pH control measures were not implemented in the first batch of the cultivation system, active pH management was adopted during the second batch.

#### 2.8.4. Agitation Speed and Impeller Type

The agitation speed was set at 200 rpm, and a Rushton turbine impeller was used in the bioreactor. This impeller type was selected for its efficient mixing capabilities, ensuring uniform distribution of nutrients and oxygen throughout the culture medium, thereby enhancing bacterial growth and metabolite production.

#### 2.8.5. Foam Control

To regulate foam formation during batch fermentation, antifoam oil (silicon oil) was added through an antifoam probe located on the head plate. This helped prevent excessive foam accumulation, which could interfere with mixing and oxygen transfer in the bioreactor.

#### 2.8.6. Analytical Measurements

Throughout the cultivation period, samples were periodically collected through a sampling system positioned at the lower section of the vessel. These samples were analyzed for various parameters, including total carbohydrates, dry weight, residual carbohydrate concentration (gL^−1^), nitrogen content, and cell biomass (cell dry weight; gL^−1^). These analytical measurements provided insights into the growth kinetics and metabolite production profiles of the bacterial culture under different cultivation conditions. 

The following equation was used to calculate the percentage yield of bacteriocin:

(1) $$Yield\;(\%)=\frac{bacteriocin\;(g)}{CDW\;(G)}\times 100$$

The volumetric bacterial biomass productivity is measured via the following equation:

(2) $$Productivity\;({gL}^{-1}h^{-1})=\frac{CDW\;({gL}^{-1})}{Time\;(h)}$$

The bacteriocin productivity was detected using the following equation:

(3) $$Productivity\;({gL}^{-1}h^{-1})=\frac{Bacteriocin\;({gL}^{-1})}{Time\;(h)}$$

##### Quantification of Total Carbohydrates

A 1 mL sample of the supernatant was diluted 500-fold using distilled water. This was mixed with 5 mL of 0.2% Anthrone reagent and chilled in ice for up to 5 min. before heating in a boiling water bath for up to 11 min. After cooling to room temperature for 60 min, the sample was analyzed at 620 nm using a BIO-RAD SmartSpecTM3000 spectrophotometer. Carbohydrate concentrations were derived from a standard curve using glucose as the standard.

##### Evaluation of Total Nitrogen

Total nitrogen levels, encompassing both organic and inorganic forms, were analyzed using the Kjeldahl method at the Microanalytical Center King Fahd Center-KAU.

##### Measurement of Cell Dry Weight

A 5 mL aliquot from each sample was filtered through Whatman filter paper (Whatman No. 1, 11 μm) and subsequently dried in an oven at 70 °C until a constant weight was achieved. The weight of the dried cells was calculated by determining the weight difference before and after filtration.

## 3. Results

### 3.1. Bacterial Isolation and Identification

**Figure 1:**
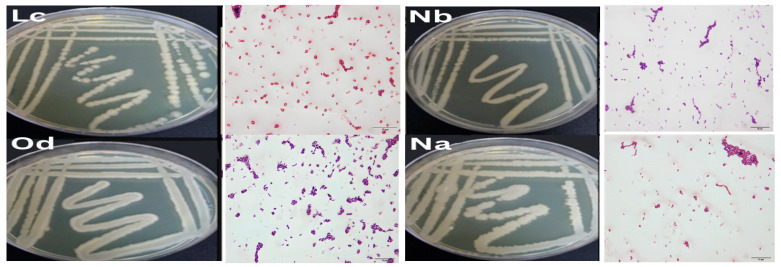
Colony morphology and Gram staining of the four most potent bacteriocin-producing *Bacillus isolates* from soil samples, characterized on nutrient agar plates.

**Figure 2:**
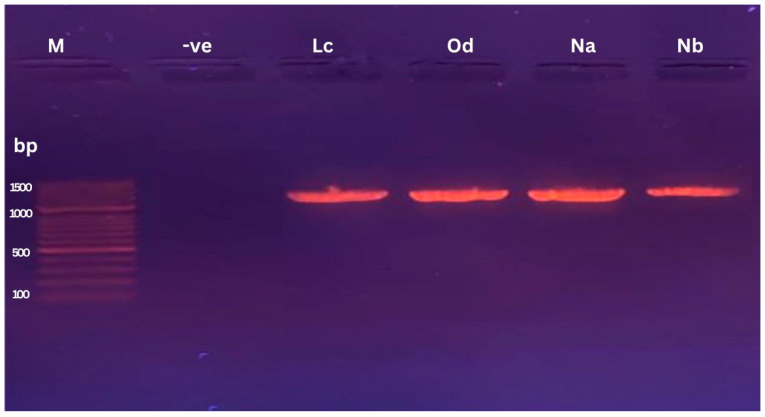
Agarose gel electrophoresis showing the amplified PCR products of isolated bacteria with a molecular weight marker (100 bp ladder). Lane M, DNA marker (GeneDirex, Inc.); lanes Lc, Od, Na, Nb are 16S rRNA samples.

**Figure 3:**
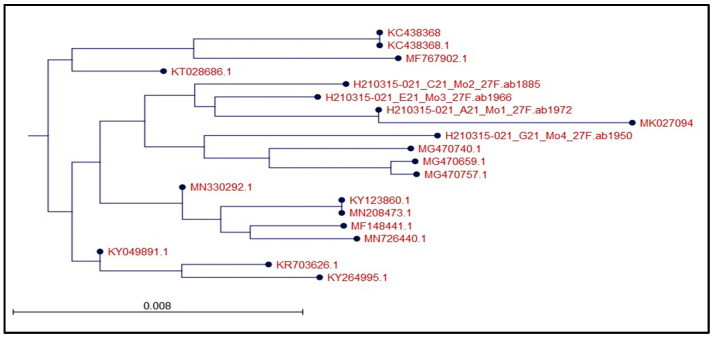
Phylogenetic tree based on the 16S rDNA region alignment of *Bacillus subtilis* (MO1-27F), *Bacillus amyloliquefaciens* (MO2-27F), *Bacillus subtilis* (MO3-27F), and *Bacillus atropaeus* (MO4-27F) with other *Bacillus* spp. accessions on Genbank. The dendrograms were obtained by neighbor-joining (NJ) using CLC Main Workbench V8.1.3 (Qiagen, Bioinformatics, Hilden, Germany). The numbers at the branch nodes represent the bootstrap values.

We successfully isolated fifteen bacterial strains from agricultural and contaminated soil samples obtained from various locations in Jizan and Jeddah. These isolates were screened for their antagonistic activities against multiple bacterial strains, including *Pseudomonas aeruginosa* ATCC 9027, Methicillin-resistant *Staphylococcus aureus* (MRSA) ATCC 33591, *Salmonella typhimurium* ATCC 14028, carbapenem-resistant *E. coli*, and MRSA 2. Two promising isolates were identified as *Bacillus atrophaeus* and *Bacillus amyloliquefaciens* based on the 16S rDNA analysis, morphological features, and microscopic examination. The microscopic analysis showed that these bacteria have rod-shaped cells with thick cell walls and can exist as single cells, short chains, or small clumps (Figure 1). The 16S rRNA genes were successfully amplified from these cultures post-DNA purification (Figure 2), with fragment sizes near 1500 bp. Sequence alignments revealed 97 to 100% similarity with other *Bacillus* species in the NCBI database (Figure 3).

### 3.2. Optimization of Bacteriocin Production

#### 3.2.1. Impact of Various Incubation Conditions on Bacteriocin Activity

**Figure 4:**
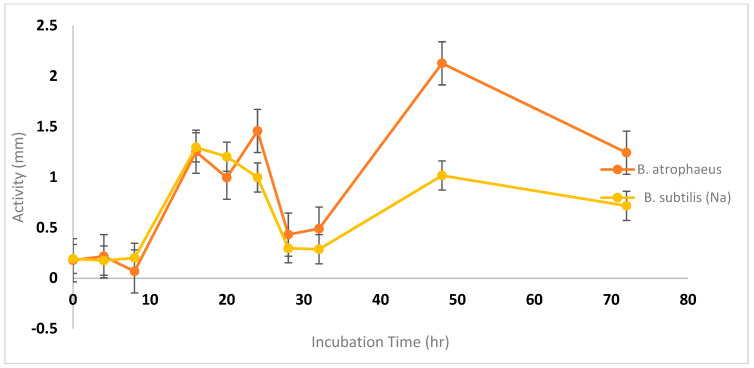
Effect of incubation time on the production of cell-free supernatants (CFSs) from *Bacillus atrophaeus* (Lc) and *Bacillus amyloliquefaciens* (Nb).

**Figure 5:**
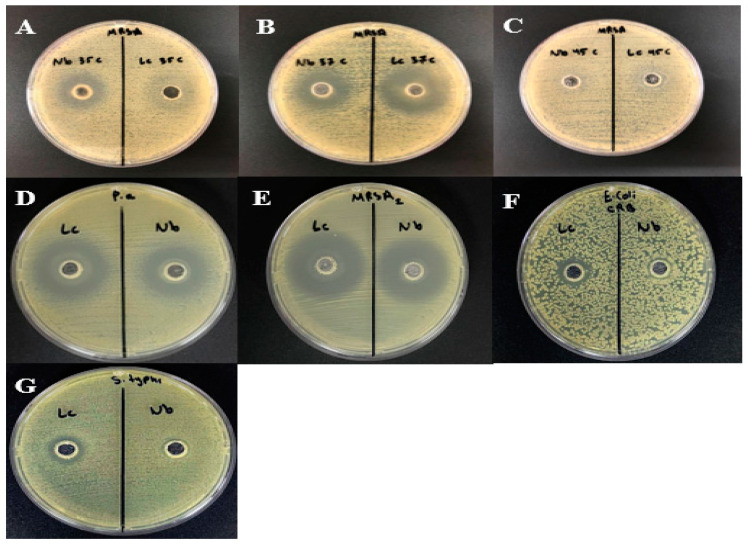
Evaluation of antimicrobial activity of the bacteriocin against selected bacteria at different incubation temperature by agar well diffusion assay. Nb refers to the bacteriocin produced by *Bacillus amyloliquefaciens.* Lc refers to the bacteriocin produced by *Bacillus atrophaeus.* (**A**) 35 °C, (**B**) 37 °C, (**C**) 45 °C. (**D**) Effect of Nb and Lc bacteriocin on *Pseudomonas aeruginosa* at 37 °C. (**E**) Effect of Nb and Lc bacteriocin on MRSA2 at 37 °C. (**F**) Effect of Nb and Lc bacteriocin on carbapenem-resistant *E. coli* at 37 °C. (**G**) Effect of Nb and Lc bacteriocin on *Salmonella typhimurium* at 37 °C.

The antibacterial effectiveness of cell-free supernatants (CFSs) from *Bacillus atrophaeus* and *Bacillus amyloliquefaciens* was tested against MRSA using an agar well diffusion assay. As the incubation time increased, we observed a corresponding expansion in the inhibition zones. For instance, the inhibition zones ranged from 13 to 24 mm for *Bacillus atrophaeus* and 15 to 24 mm for *Bacillus amyloliquefaciens* over a 72 h period (Figure 4). The temperature-dependent assays showed that *Bacillus amyloliquefaciens* (Nb) had greater antibacterial activity at 35 °C compared to *Bacillus atrophaeus* (Lc), but both exhibited similar levels of activity at 37 °C. Remarkably, both strains demonstrated negligible antibacterial activity at 45 °C. At 37 °C, both strains displayed significant antibacterial activity against *Pseudomonas aeruginosa* and MRSA but had a minimal effect on carbapenem-resistant *E. coli* and *Salmonella typhimurium* (Figure 5).

**Figure 6:**
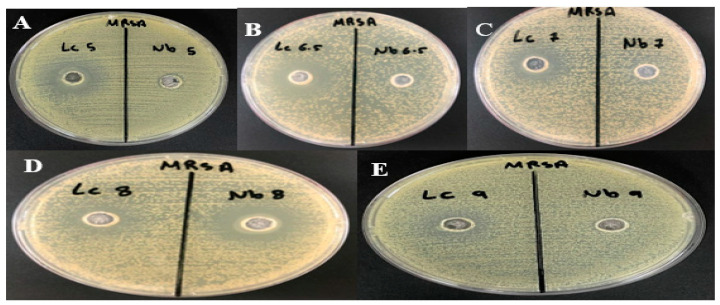
Evaluation of antimicrobial activity of the bacteriocin against selected bacteria at different pH values by agar well diffusion assay. Nb refers to the bacteriocin produced by *Bacillus amyloliquefaciens.* Lc refers to the bacteriocin produced by *Bacillus atrophaeus.* (**A**) pH 5.0. (**B**) pH 6.5. (**C**) pH 7.0. (**D**) pH 8.0. (**E**) pH 9.0.

In the pH-dependent assays, *Bacillus atrophaeus* (Lc) demonstrated higher antibacterial activity than *Bacillus amyloliquefaciens* (Nb) at pH levels of 5.0, 6.5, and 7.0. Conversely, Nb displayed stronger antibacterial activity at pH 8.0 (Figure 6).

#### 3.2.2. Effect of Different Growth Media on Bacteriocin Activity

**Figure 7:**
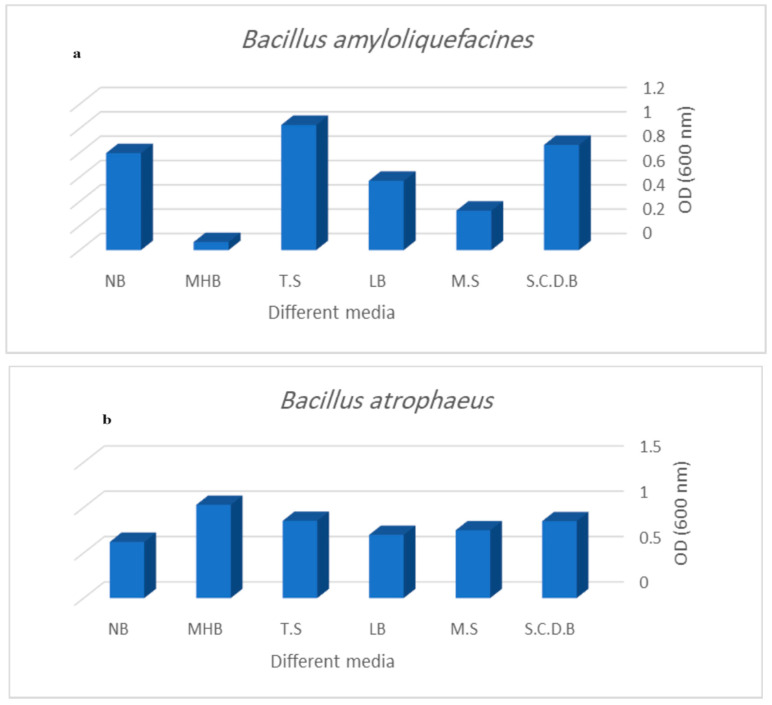
Evaluation of antimicrobial activity of (**a**) *Bacillus amyloliquefaciens* and (**b**) *Bacillus atrophaeus* against selected bacteria at different fermentation medium by agar well diffusion assay.

The growth and bacteriocin production rates were assessed in different fermentation media (Figure 7a,b). MHB was found to be optimal for *Bacillus atrophaeus*, while TSB was best suited for *Bacillus amyloliquefaciens*.

### 3.3. Impact of pH Control on Cell Growth and Bacteriocin Production

**Figure 8:**
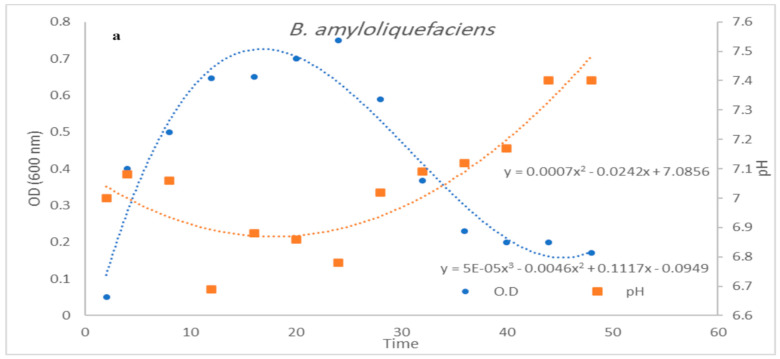

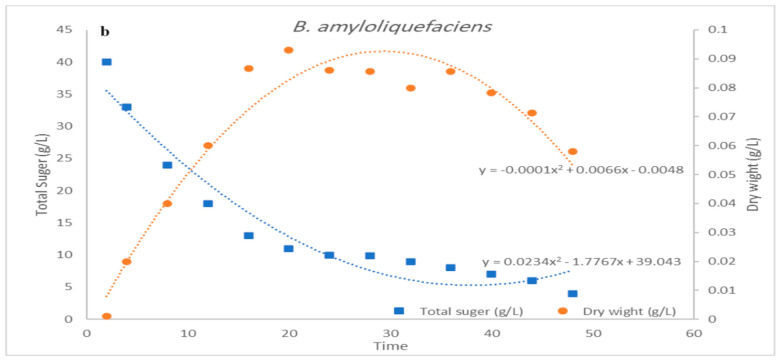
(**a**,**b**) Effect of batch cultivation process at 150 agitation, 1 (*v*/*v*) aeration at 35 °C on *Bacillus amyloliquefaciens* growth O.D 600 nm, cell dry weight (gL^−1^), and substrate consumption under (**a**) uncontrolled and (**b**) controlled pH conditions.

**Figure 9:**
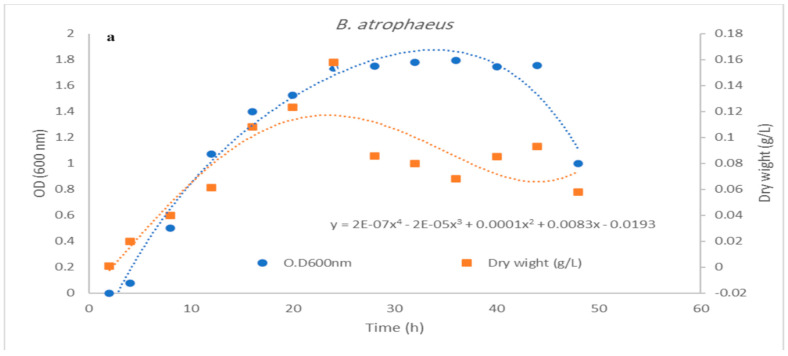

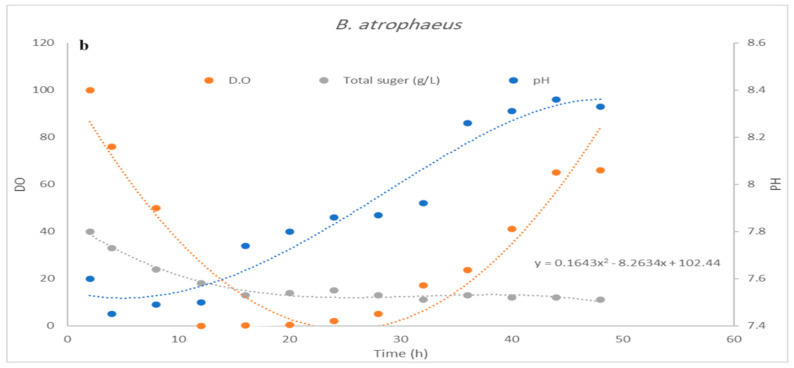
(**a**,**b**). Effect of batch cultivation process at 150 agitation, 1 (*v*/*v*) aeration at 35 °C on *Bacillus atrophaeus* growth O.D 600 nm, cell dry weight (gL^−1^), and substrate consumption under (**a**) uncontrolled and (**b**) controlled pH conditions.

Both controlled and uncontrolled pH conditions were explored in a 10 L stirred tank bioreactor (Figure 8a,b and Figure 9a,b). Controlled pH conditions led to a notably higher average cell growth rate (0.21 gL^−1^ h^−1^) compared to uncontrolled conditions (0.11 gL^−1^ h^−1^). Moreover, the bacteriocin yields were higher in controlled conditions, peaking at 16.05 gL^−1^ compared to 11.3 gL^−1^ under uncontrolled conditions. Interestingly, the controlled pH conditions produced approximately 20% more bacteriocin (1.02 gL^−1^ h^−1^) than the uncontrolled conditions (0.8 gL^−1^ h^−1^). When evaluating the bacteriocin production yield coefficient (Y_P/X_), the maximum yield achieved under the controlled conditions was nearly double that achieved under the uncontrolled conditions. These results suggest that pH control can significantly influence both the cell growth and bacteriocin production rates. In summary, our experiments showed that controlling the incubation conditions and growth medium can profoundly impact the antibacterial efficacy and yield of bacteriocin production. This is particularly evident when comparing bacteriocin yields and bacterial growth in flask cultivations versus batch process cultivations. The batch process method yielded significantly higher cell densities and bacteriocin production rates, primarily because it prevented nutrient limitations often encountered in flask cultivations.

## 4. Discussion

The production of secondary metabolites, especially antibiotics, by bacteria holds significant importance both commercially and for research purposes. The increasing prevalence of antibiotic-resistant bacteria due to widespread antibiotic usage underscores the need for screening new antimicrobial agents [29]. In this study, we successfully isolated antimicrobial compounds from two *Bacillus* strains obtained from agricultural and polluted soil samples. These bacterial isolates were identified as *Bacillus amyloliquefaciens* and *Bacillus atrophaeus* through morphological, microscopic, and 16S rDNA gene sequencing analyses. These findings are consistent with those reported by [30]. Prior studies have also documented the isolation of antimicrobial compounds from *Bacillus atrophaeus*. For instance, ref. [31] identified an antimicrobial compound from *Bacillus atrophaeus* found on frog skin, while [32] isolated a strain of *B. atrophaeus* from marine sponges that produced an anti-algal compound known as Beclamide C. Additionally, ref. [33] indicated that most *Bacillus* species are bacteriocin producers and have a long-standing history of safe usage in the food industry, with antibacterial properties effective against a variety of pathogenic bacterial strains. Our study revealed that the antimicrobial compounds extracted from the two *Bacillus* species demonstrated broad-spectrum antibacterial activity against both Gram-negative and Gram-positive bacteria. Compounds Lc and Nb showed particularly strong activity against Gram-positive bacteria like MRSA, as well as Gram-negative bacteria including *E. coli*, *P. aeruginosa*, and *S. typhimurium*, corroborating findings by [31,32,33]. The exploration of multiple bacteriocins from different species provides a more comprehensive understanding of their potential applications in antimicrobial therapy and other fields. This approach harnesses the natural diversity of antimicrobial peptides and maximizes the chances of discovering effective solutions to combat microbial pathogens [34]. The selection of different growth media for testing bacteriocin production in *Bacillus* species was based on several considerations aimed at optimizing the production process. Mueller–Hinton broth was chosen for its low nutrient content, making it suitable for assessing the true antibacterial activity of bacteriocins in *Bacillus atrophaeus* [35]. Conversely, Luria broth provides a rich nutrient composition, potentially promoting higher levels of bacteriocin production due to the availability of complex nutrients [36]. Tryptic Soy broth offers a balanced nutrient composition, supporting the growth of a wide range of bacteria and facilitating optimal bacteriocin production in *Bacillus amyloliquefaciens* [37]. Brain Heart Infusion was included for its nutrient-rich formulation, which may enhance bacteriocin production, particularly in *Bacillus* spp., which require higher nutrient levels [38]. Soybean Casein Digest broth provides a rich source of amino acids and peptides, potentially supporting enhanced bacteriocin production [39]. Nutrient broth offers a general-purpose medium with essential nutrients for bacterial growth, suitable for evaluating bacteriocin production under standard laboratory conditions [40]. Mineral Salt Medium was selected as a minimal medium containing inorganic salts and a single carbon source, providing a controlled environment for studying bacteriocin production in the absence of complex nutrients [41]. By testing bacteriocin production in various growth media with different nutrient compositions, we aimed to evaluate the influence of factors such as carbon and nitrogen sources on bacteriocin synthesis. This comprehensive approach helps identify optimal conditions for maximizing bacteriocin yields, which is crucial for potential applications in antimicrobial therapy and food preservation. Optimal antibacterial compound synthesis occurred at 37 °C and a pH of 7.0. Interestingly, our bacterial isolates were versatile enough to produce antibacterial compounds across a temperature range of 35–37 °C. The isolated compounds remained stable and active over a wide pH range, from 4.0 to 9.0. This contrasts with other newly described antimicrobial peptides, which tend to lose activity under very acidic conditions and show a steady decline in effectiveness at basic pH levels. Our results align with those of Naclerio et al. [18], although their optimal production conditions differed, occurring at 25 °C and a pH of 7.0. The antibacterial activity of peptides Lc and Nb derived from *Bacillus atrophaeus*, *Bacillus amyloliquefaciens*, and *Bacillus subtilis* demonstrated variable inhibitory zones against MRSA at different incubation times at 37 °C. The inhibition zones for the two *Bacillus isolates* expanded with increased incubation time up to 72 h. Overall, bioreactor-based cultivation exhibited a superior performance compared to shake flask cultivation. This can be attributed to the more favorable growth conditions provided by the bioreactor, including better aeration, agitation, and nutrient distribution, leading to enhanced bacterial cell productivity.

## 5. Conclusions

This study has provided valuable insights into the isolation and characterization of antimicrobial compounds produced by *Bacillus* strains obtained from agricultural and polluted soil samples. This study revealed the broad-spectrum antibacterial activity of these compounds, showing effectiveness against both Gram-positive and Gram-negative bacterial strains. Notably, the antimicrobial compounds remained stable and effective across a wide range of environmental conditions, including various temperatures and pH levels. These findings not only corroborate the existing literature but also extend our understanding of the potential applicability of *Bacillus*-derived antimicrobials, particularly in an era where antibiotic resistance is a mounting concern. Additionally, our comparative analysis between the bioreactor and shake flask cultivation methods demonstrated a superior efficacy to the former. The bioreactor conditions led to improved bacterial growth and higher antimicrobial compound yields, likely due to optimized aeration, agitation, and nutrient distribution. These results suggest that scaling up to bioreactor-based production could be a feasible and more effective approach for commercial applications.

## Data Availability

All authors declare that the data supporting the findings of this study are available within the article.
